# Optical Active Meta-Surfaces, -Substrates, and Single Quantum Dots Based on Tuning Organic Composites with Graphene

**DOI:** 10.3390/ma17133242

**Published:** 2024-07-02

**Authors:** Marcelo R. Romero, A. Guillermo Bracamonte

**Affiliations:** 1 Departamento de Química Orgánica, Facultad de Ciencias Químicas (Universidad Nacional de Córdoba), IPQA−CONICET, Córdoba CP 5000, Argentina; marcelo.ricardo.romero@unc.edu.ar; 2 Departamento de Química Orgánica, Facultad de Ciencias Químicas, Instituto de Investigaciones en Físicoquímica de Córdoba (INFIQC), Universidad Nacional de Córdoba, Ciudad Universitaria, Córdoba CP 5000, Argentina

**Keywords:** graphene optical properties, chemical surface modifications, optical substrates, optical active metasurfaces, tuning of non-classical light, nanodevices, microdevices

## Abstract

In this communication, the design and fabrication of optical active metamaterials were developed by the incorporation of graphene and joining it to different substrates with variable spectroscopical properties. It focuses on how graphene and its derivatives could generate varied optical setups and materials considering modified and enhanced optics within substrates and surfaces. In this manner, it is discussed how light could be tuned and modified along its path from confined nano-patterned surfaces or through a modified micro-lens. In addition to these optical properties generated from the physical interaction of light, it should be added that the non-classical light pathways and quantum phenomena could participate. In this way, graphene and related carbon-based materials with particular properties, such as highly condensed electronics, pseudo-electromagnetic properties, and quantum and luminescent properties, could be incorporated. Therefore, the modified substrates could be switched by photo-stimulation with variable responses depending on the nature of the material constitution. Therefore, the optical properties of graphene and its derivatives are discussed in these types of metasurfaces with targeted optical active properties, such as within the UV, IR, and terahertz wavelength intervals, along with their further properties and respective potential applications.

## 1. Introduction

This communication discusses the design of metamaterials for light tuning in different wavelength intervals and applications. The optical active metamaterials discussed are based on the following: (i) modified photonic surfaces, (ii) modified confined paths of optical substrates, and (iii) single nano-metamaterial platforms. In this manner, tunable properties from the interaction of controlled sources of light of different natures could be used for different applications. Photoactive surfaces for biosensing, energy storage, and electronic conduction could be developed from modified surfaces. Meanwhile, from modified substrates, new non-classical light pathways could be generated using metamaterials with new optical uses, such as optical active lenses with specific optical functions such as light polarization, absorption, and tunable wavelengths. These mentioned optical functionalities could be incorporated into devices such as nanodevices, microdevices, waveguides, and chips. It is noted that metamaterials and metasurfaces are related, with matter compositions that produce different properties in comparison to the sum of the individual characteristics provided by individual optical components. Thus, optical platforms and devices could be considered, like subwavelength arrays of optical scatters, which could produce different light pathways. From these devices, optical active metamaterials could be produced, such as augmented lenses, crystals, and confined paths from enhanced light interactions within modified substrates and surfaces. In this manner, superlenses operating at visible wavelengths were developed for imaging applications [1]. Thus, improved resolutions were achieved by light tuning below the limit of refraction with the use of a lens for imaging applications. Light tuning was based on the interactions of photons within hole arrays through GaP cores on a sapphire substrate protected with silica, such as optical active waveguides that generated light shifts and interactions. In this manner, the resolution of the generated images was increased. 

In a similar manner, the modification of surfaces with controlled nano-patterning could lead to light pathway modifications and improved performances in imaging resolution. Thus, miniaturized optical components based on metamaterials could be incorporated within different optical setups, such as micro-lenses, filters, and functional imaging platforms [2].

For the development of optical active metamaterials, different types of nanomaterials with variable properties could be incorporated to combine new properties explained by their interactions with electromagnetic fields, photons, electrons, and other energy modes. Moreover, in the well-defined structures of graphene, there is a two-dimensional crystal with a zero bandgap and a zero effective mass of charge carriers [3]. Moreover, graphene is like a typical monolayer of carbon in a hexagonal lattice that could interact with other layers, modifying their intrinsic electronic densities. Thus, it was highlighted that even within these scales, the mentioned phenomena showed a high sensitivity against the size scaling following quantum physics laws. Thus, these apparent invisible laws affected the nanoscale and microscale optics. The synthesis of graphene quantum dots permitted by well-controlled organic chemistry reactions showed the versatility of varying sizes, stabilizing agents, and chemical surface modifications. In this manner, variations in electron mobility are afforded to different photonic and photovoltaic applications. Moreover, graphene shows interesting semiconductive properties with high-impact studies and applications such as within the nano- and microelectronics perspectives, electron harvesters, and energy applications [4,5]. As a practical example of the effect of graphene on conductive materials, the enhancement of copper/graphene composites based on high-quality graphene is noted [6]. A nearly 10% higher conduction was afforded compared to that with copper only, in addition to noting an increased hardness of the melted composite. These properties resulted from better electronic conduction through hybrid material and cohesion between both components [7]. Moreover, it highlighted the capability of graphene and derivatives to produce pseudo-electromagnetic fields [8] generated from their high electronic conjugation. The near field could interact with its surroundings, modifying its electronic [9] and optical properties. For example, theoretical calculations of multi-hole arrays on graphene metasurfaces recorded the generation of enhanced electromagnetic fields from the interaction of the confined electronics of graphene holes [10]. In this manner, a wide topological bandgap at terahertz frequencies with the application of a low pump power of less than 10 nW was recorded. These types of studies could show potential uses by their incorporation within optical surfaces and substrates. In this manner, graphene could develop new optical active materials in variable intervals of wavelengths and frequencies such as ultra-violet (UV) [11], infra-red (IR) [12], terahertz [13], and further absorptions [14,15]. In this context, it should be noted that graphene showed optical active behavior within different intervals of wavelengths based on different phenomena involved, for example, 2D semi-metal/conductive sheets, where only light polarized parallel to graphene’s plane can effectively induce surface currents and Ohmic optical absorption [16]. Meanwhile, terahertz signals were produced from transitions of graphene’s intrabands [17], and near NIR and visible absorptions were dominated by interband transitions [18]. Therefore, variations in the basis of the phenomena produced should be mentioned in order to consider them in the design and synthesis.

These examples were important in the presence of tuned graphene metamaterials; however, joining other nanomaterials could couple their properties to generate new ones. In this context, the formation of hybrid core–shell nanoplatforms was highlighted by joining gold core templates modified with graphene that produced enhanced plasmonic properties with higher electrochemical sensitivity for targeted molecular detections [19]. In a similar manner, we highlight the enhancement of evanescent waves near the surface of a prism coated with several layers of metal, graphene, and double-negative materials [20]. The thicknesses of the metallic and double negative films were tuned with a controlled number of graphene layers to record the maximal highest enhancement factor. Thus, graphene has a direct implication in the generation of increasing evanescent waves. The energies produced were proposed for optical trapping applications so they could be tuned for other types of optical developments and applications. 

In this regard, it is important to note how easily graphene and its derivatives could be obtained and how important purity is to develop optimal performances. This article does not intend to describe synthetic methods, but this important topic is mentioned considering the varied sources of carbon. In this manner, the chemistry of surfaces could vary, adding versatility to the joining of different optical active materials within varied scales. Recently, a new solubilizing strategy was developed that led to the synthesis of stable colloidal graphene quantum dots with more than 100 conjugated carbon atoms, allowing the study of their properties in a new size regime. In this perspective, variations in their energy relaxation dynamics have recently been highlighted. In particular, it was observed how unconventional slow “electron cooling” related to the relaxation of electrons from high excited states to lower ones. These high-energy electrons could potentially be harvested in solar energy applications. At this level, the modifications from the source of carbons to chemical surface passivating agents are variables that affect quantum phenomena considering the interactions of 100 confined carbon atoms. The innovative synthesis of carbon-based materials from carbon dots of varied sizes to quantum dots is used to manage carbon sources and energy to break and form covalent bonds. In this regard, hydrothermal processes are permitted to manage aqueous media and energy delivery to accomplish the synthesis. Combining highly conjugated carbon-based molecules such as Rhodamine B and o-Phenylenediamine and similar molecules with bifunctional linkers could be achieved—carbon-based materials, for example. The use of o-phenylenediamine with varied precursors and changes in the reaction media was afforded to N-doped carbon nanodots (N,S-doped in case of thiourea addition) with less than 10 nm spherical diameters [21]. Further examples are developed based on this simple strategy of chemical modification that opens up carbon-based materials to be used within aqueous media. In order to have an idea about the efficiency of emitters that could be developed by this synthetic pathway, orange emitting carbon dots (CDs) were synthesized by a hydrothermal process, showing strong and stable emissions at 574 nm by 555 nm laser excitation with a fluorescence quantum efficiency of 46% [22].

Then, in order to highlight the importance of the varied sources with different structures, it is mentioned how reduced sizes of dots, such as graphene quantum dots, and varied sources of selection of intrinsic structures could influence the product obtained. Graphene can be generated with the incorporation of other heteroatoms within the 2D surface or covalently linked on their surfaces. In this way, recently, it was shown that the synthesis of graphene quantum dots using bio-waste carbon-based material sources to produce amine-alfa-terminated GQDs (AmαGQDs), which have higher dispersibility and photoluminescence intensity than those of GQDs in the absence of amine groups and further passivating agents provided a complex natural source. 

Therefore, graphene could modify its photophysics, highlighting the excited state as well as its optoelectronic properties based on their high conductive layers and condensed electronic orbitals with electromagnetic fields perturbing electronics in their surroundings, generating new spectroscopical characteristics and consequent optical properties depending on the joined nanomaterials [23]. In this manner, the optics could be specialized for different purposes depending on the optical material design, the optical stimulation applied, optical setup, and instrumentation used.

**Figure 1:**
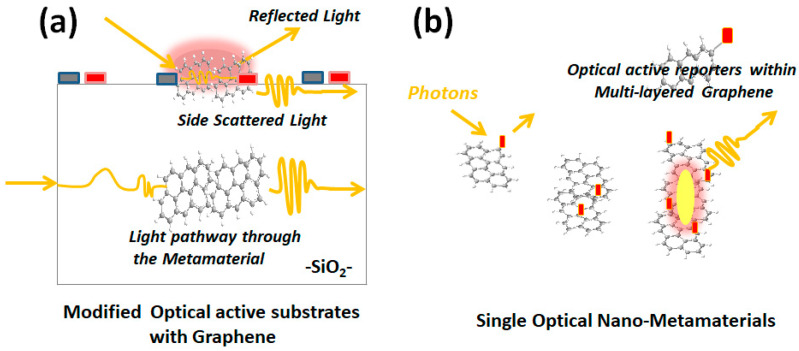
Schema of photon interactions in different optical approaches: (**a**) across and through modified optical active substrates with graphene and (**b**) single optical nano-metamaterials with the incorporation of multi-layered graphene. Reprinted with permission of A. Guillermo Bracamonte et al. [23].; Copyright *Bentham Sci.* 2020.

For these reasons, this review article discusses the different experimental approaches exploiting the particular properties of graphene. In this manner, their accurate incorporation in bulk materials as metamaterials to interact and modify the light pathway was discussed (Figure 1a). These types of metamaterials could be incorporated within devices, lenses, and miniaturized instrumentation. Moreover, recent advances in nano-optics from the developments of single nano-metamaterial platforms by the incorporation of graphene quantum dots are discussed (Figure 1b). These types of developments show different interests within confined nanodevices as well as toward the micro- and macroscale. However, the mention and discussion of single-dot analysis or nanospectroscopy were intended to develop the capability and control of non-classical light from the atomic and molecular interactions to the close surroundings within nanoscale lengths.

In a similar manner, these focused optic developments could be used for other types of studies and applications than bulk materials. For example, quantum circuits, chips, quantum emitters, and nanodevices toward microdevices with multi-wavelength responsive optical dots, etc. For all these developments and varied materials, different methodologies should be considered, such as wet chemistry and laser-assisted techniques [24].

In this manner, independently of the approach developed, it should be mentioned briefly that graphene, due to its intrinsic chemical constitution, could be applied as an energy absorber or emitter of electromagnetic fields and quantum energies depending on the studied properties. From this perspective, it should be noted that in the last 5 years, the number of publications related to the generation of optically active materials increased exponentially. This trend was based on the demand of the market. Thus, fundamental knowledge from researchers at the top of the design and fabrication manufacturers produced the right technological transfer required. Moreover, this fact notes the need for the development of knowledge associated with real applications that are highly appreciated and stimulated to improve human well-being.

Therefore, in this review, we present a selection of recently reported high-impact studies and developments. The criteria for the selection of publications discussed were based on the optical setup developed by chemical modification of solid substrates and surfaces. This opened the strategy to stimulate optical active materials with high-power laser sources and varied detectors depending on the emission wavelength and mode of energy generated. Thus, light was delivered on the surface and passed through the material, and, depending on their composition, it produced varied signaling with high-impact perspectives for multi-modal approaches. In this regard, the articles presented afforded optical metamaterial designs with the incorporation of graphene and derivatives.

## 2. Photonics from Metasurfaces and Metamaterials Based on Graphene within Variable Intervals of Wavelengths 

The tuning of photons from light–matter interactions, considering not only the power of irradiance from varied natural and technological light sources, is of high impact and interest due to the attention paid to the created material. Graphene and its highly condensed electronic properties showed important fundamental research insights that could lead to the next generation of technology. In the next subsections, we highlight these important facts, further future developments, and some of the most well-known and used methodologies in order to tune optics considering graphene and its derivatives.

Different strategies and methods exist to manipulate optical active materials towards varied architectures, for example, 2D monolayers, top-down exfoliation methods, such as mechanical and liquid-phase exfoliation, and bottom-up growth methods using chemical vapor deposition (CVD) and related approaches [25].

There is an interest in controlling 2D structures from a broad overview of high-impact research, where the capability provided by non-covalent interactions is highlighted. Thus, 2D materials are easily formed, permitting the production of libraries of photonic structures, such as integrated waveguides, optical fibers, photonic crystals, and metasurfaces. In these materials, 2D multi-layered graphene is incorporated by van der Waals interactions [26]. This non-covalent bonding enabled the joining of varied materials with accurate templated topographies, such as holed, porous, and nano-patterned materials. In this manner, multi-layered graphene can participate in resonant and enhanced physical and chemical pathways. Moreover, it is noted that 2D materials enable handiness in transfer and mixing with various prefabricated photonic templates with high degrees of freedom, improving optical gains, enhancing signaling, modulating light pathways, sensing, and developing new plasmonic modes. In addition, it is noted that 2D materials via chemical or physical engineering dramatically augment light–matter interactions, leading to the enhanced performance of existing functional devices explained by new metaphotonic phenomena [27]. Other techniques and methods, such as spin-coating techniques applied to homogeneous deposition of varied semiconductors, are well known and permitted to produce varied optically active photomasks and optically active surfaces. For example, spin coating could be used for rational adjustment to interfacial interactions of carbonized polymer dots with efficient large-area perovskite light-emitting diodes [28].

Additional methods could be adapted to modify substrates. For example, photo-polymerization can be used to develop printed surfaces with varied optically active components covalently linked within complex polymeric structures [29]. Finally, there are advanced new methodologies in the design of experiments and methods contemplating theoric calculations. For example, finite element method (FEM) simulations were largely employed to increase the understanding of phenomena related to the generation of polymer sheets where varied materials could be incorporated [30]. Thus, from the concept to the methodology and fabrication, new types of composites for varied studies and applications could be proposed.

Thus, the high-impact developments towards new perspectives contemplating the properties and versatility of graphene could be discussed depending on the varied targeted uses.

### 2.1. Graphene-Based Absorber Metamaterials

From the basis of graphene absorption, the spectroscopic properties are based on highly conjugated carbon chemical structures and different stretching modes with UV, near-, and far-IR absorptions and further frequencies. Thus, graphene could be used as a potential absorber within large wavelength intervals to tune new matter properties, where metasurfaces and new matter properties could be generated.

For example, from controlled multi-layered graphene synthesis by an exfoliative oxidation reaction, a variable multi-layered graphene composite with different absorbance properties was produced. The augmented absorbances were based on the inter-pseudo-electromagnetic field’s interactions between the variable inter-layer interactions with the consequent generation of varied absorbers species [31]. Another example in the IR interval of wavelength from the theoretical calculation of reduced graphene oxide [32] showed variations in the IR absorptions and emissions depending on the defects and functional groups attached. In this manner, it was recorded that reduced graphene oxide prepared by Hummers’ method was strong in the near- and far-IR bands but weaker in the middle IR band. Therefore, reduced graphene oxide can be joined and coupled to other compatible materials.

**Figure 2:**
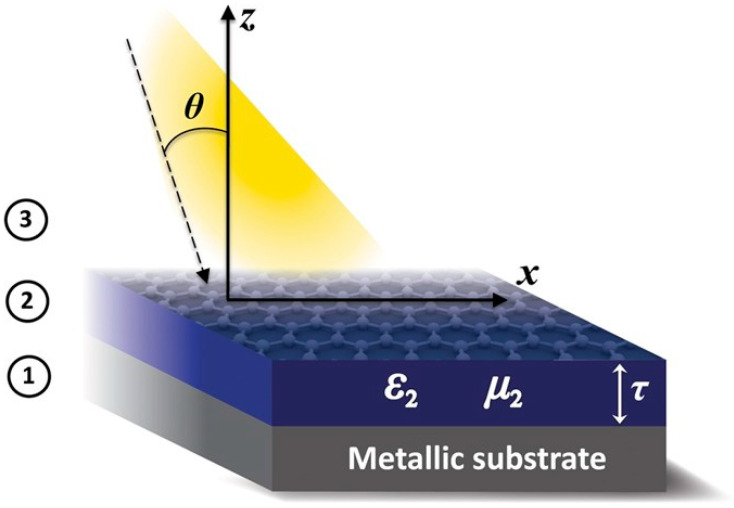
Considered setup: an EM wave in vacuum (region 3) is incident upon a graphene layer coating an anisotropic ENZ metamaterial (region 2). A perfectly conducting metal backs the entire structure (region 1). Reprinted with permissions of K. Halterman et al., 2016 [33], Scientific Reports, Springer Nature.

In this manner, by the accurate deposition of graphene on metallic surfaces, a near-perfect absorption was observed that arose over an unusually broad range of beam incidence angles (Figure 2) [33]. Due to the presence of graphene, the absorption was tunable via a gate voltage, providing dynamic control of the energy transmission. Thus, it was shown that this strongly enhanced absorption arises due to graphene–metallic coupling, generating new and fast electromagnetic wave-mode propagations along the graphene/metamaterial hybrid achieved. In this research work, the solutions of the Maxwell equations are well described in the presence of graphene; however, experimental data contemplate the different methods of adding graphene to metallic surfaces. Thus, the application of varied dilutions related to different graphene concentrations could produce deposition on the surface. However, interactions between metallic surfaces and graphene are dependent on both. Therefore, these considerations should be contemplated in the next generation of modified surfaces accompanied by theoretical support.

Moreover, by accurate separation of metallic graphene with dielectric spacer incorporation, tunable IR absorption peaks were obtained from theoretical simulations controlled by changing the Fermy Energy level of graphene and geometry parameters of the setup design [34]. In this manner, high absorption could be predicted at a wide range of incident angles. This device could permit tunable sensors, filters, detectors, or other graphene-based photonic devices. In addition, there is a possibility of periodic arrays as graphene stack metamaterials formed by unit cells with graphene acting as a thin insulating spacer, which allows for the accumulation of strong absorption from individual graphene sheets. Moreover, low reflectivity from the stack material and high absorption properties showed potential applications for optoelectronics devices [35].

### 2.2. Metasurfaces and Modified Substrates for Non-Classical Light and Emissions Tuning

The 2D electron conductivities with quantum implications and 3D electromagnetic waves in graphene metamaterials could be used to tune electron optics properties [36]. In this manner, first, we mention the synthesis of meta-graphene surfaces with an accurate thickness of 90 nm that permits 85% absorptivity of unpolarized light [37] in the visible and near-infrared interval of wavelengths. The meta-material consisted of the alternation of graphene and dielectric shields that acted as light waveguide modes to achieve broadband absorption over incident angles up to 600. Thus, potential applications for energy applications were shown, such as solar thermal uses permitted to record heating of 1600 °C with natural sunlight. 

**Figure 3:**
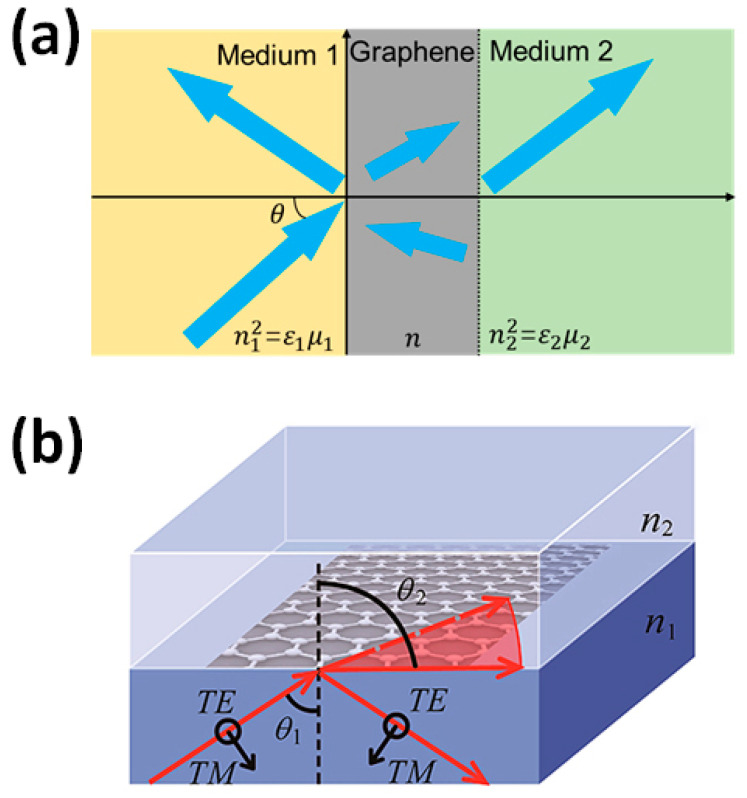
Optical metamaterial substrate with graphene incorporation. (**a**) Schematic of a graphene layer sandwiched between two dielectrics (RI, n1 > n2), (**b**) 3D design of the modified substrate with incident, refractive, and scattered light beams. Reprinted with permissions of F. Xing et al., 2020 [38], Int. J. Mol. Sci., MDPI.

The enhanced absorption could also be tuned for increased polarized optical properties. In this way, multi-layered optical substrates could be designed (Figure 3) with the incorporation of graphene [38]. Thus, dense electronic properties and plasmonics are produced based on their pseudo-electromagnetic fields that could interact with the electromagnetic vectors of light to modify and tune polarized states of light. 

In these types of meta-materials, it should be contemplated that, according to the principle of polarized light generation, traditional polarizing devices can be roughly divided into the following three categories: (i) absorptive polarization controller using material anisotropic absorption, (ii) prism polarization controller while using refraction effect, and (iii) Brewster angle polarization controller using reflection. Thus, different parameters to control could modify the final polarization properties. In this manner, graphene-based multi-layered metamaterials with phototunable architecture for on-chip photonic devices [39] could be obtained by different approaches. However, in general, most were based on modified multi-layered polymeric substrates. The contemplation of the controlled addition of multi-layers is very important for incorporating spacer lengths and for the generation of new physical and chemical properties. The inter-layer interactions could produce modifications in the electromagnetic fields of the interacting matter constitutions when they are optically active. In this manner, further interactions are affected as well. From this perspective, the design and control of accurate distances are highly expected to modify light–matter interactions and optical properties.

Other frequencies generated from graphene-based metasurfaces and metamaterials were the terahertz absorption and emissions. In this field, the design of functional films with conductive media by incorporating graphene wires to tune absorber surfaces within these frequency intervals for electronic shuttle uses should be mentioned [40]. Within these frequencies, graphene-based tunable negative refractive index metamaterials and applications in dynamic beam-tilting terahertz antennae were recently reported [41]. These types of nanomaterials could have potential applications for non-classical light detection and transduction in biodetection. The terahertz frequency tuning from the different deposition and accurate spatial deposition, such as silicon nitride substrates modified with varied multi-layered graphene deposition on gold surfaces by vapor chemical deposition (CVD), generated strong resonant plasmonic frequencies [42]. The frequencies in all cases were generated from electromagnetic fields from inter-layers of graphene that performed absorbance and emission energies within these intervals of frequencies. In this way, a direct impact on photonics metamaterials was shown due to a large range of wavelengths with potential applications for switching, mode locking, and pulse shaping; it was even shown by non-linear responses from the hybridization of graphene with other plasmonic inorganic materials [43]. In this context, the control of light trapping by voltage-gated stimulations could tune other energy modes, such as polaritons from the strong graphene–plasmon interactions. Therefore, depending on the gate geometry and applied voltage, the resulting tuned topological effects were recorded with potential applications for on-chip and ultra-compact nanophotonic waveguides and cavities [44].

In a similar manner, Fano-resonances were developed for optical sensing due to their high sensitivity against the detection of varied analytes within the nanoscale by sensing the refractive index and infrared vibrational fingerprint modifications. These properties were based on the incorporation of graphene within the design of a split-ring resonator on a reduced-sized disk conceived to reduce energy losses [45]. 

In addition, from an optical point of view, there is interest in the fabrication of optical mirrors and the development of new properties with potential applications for optical lenses. Thus, the design of metamaterial mirrors should be discussed with the incorporation of hybrid metallic nanoparticles for enhancing broadband light absorption in thin-film optoelectronic graphene devices [46].

Finally, the design of hyperbolic metamaterials, such as switchable mid-infrared frequency reflection modulations, was afforded [47]. In this manner, by modifying the number of graphene monolayers in the hyperbolic metamaterial stack, it was able to shift the plasmonic resonance up to 3.6 µm in the function of the incident light applied. Moreover, in a similar manner, photonics crystals made of alternate layers of an isotropic ordinary dielectric and a graphene-based hyperbolic metamaterial showed particular hyperbolic dispersion of frequencies, accompanied by the generation of photonics bandgaps [48]. The hyperbolic and elliptical frequencies generated were controlled by chemical potential tuning and modification of optical axes in the metamaterial. In this manner, different energy modes and frequencies were generated and controlled within high-index isotropic layers.

So, different optical metamaterial designs, such as the modification of meta-substrates, graphene within hybrid heterojunctions, laser-assisted nano-patterning designs, and controlled inter-layered graphene deposition demonstrate the generation of different and innovative optical properties not produced in the absence of accurately targeted components. In addition, to conclude, the impact on the macroscale from collective opto-electronic excitation or stimulation of variable inter-layered graphene-based metamaterials through modified substrates should be highlighted. It is noted that further phenomena could be tuned as well in a similar manner by tuning single nanoplatforms for different applications. From these perspectives, linear and non-linear models are contemplated to explain experimental data. It is important to highlight when materials are new and not well-known for their optical properties.

## 3. Optical Metamaterial Properties from Single Quantum and Optical Active Nanoplatforms

**Figure 4:**
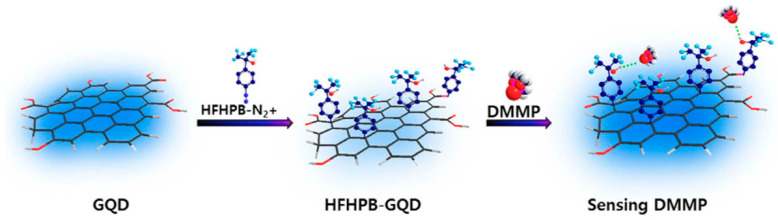
An illustration of our sensory material referred to as HFHPB-grafted GQD (HFHPB-GQD) and its interactions with the target material (DMMP). Reprinted with permissions of H. Lee et al., 2016 [52], Scientific Reports, Nature.

In this section, the use of graphene quantum dots (GQDs), chemically modified GQDs, and other high conjugated carbon-based nanoarchitectures were developed and discussed, as well as some insights by the combination of different nano-optics from varied materials. Thus, these reduced-sized GQDs could be synthesized by wet chemical methods [49] and laser-assisted techniques [50] to be used as single nanoplatforms for multiple studies, uses, and applications. Their reduced sizes, combined with their particular properties related to highly conjugated electronic densities and pseudo-electromagnetic fields below the nanoscale, could be accurately targeted by focused and short-pulsed lasers of varied excitation wavelengths [51]. From there, different types of electronics and photonic interactions could be exploited by the right tuning of their surfaces. In this way, the chemical modulation of GQDs for tuning photoluminescence for the design of novel probes and sensors could be mentioned [52]. These GQD sensor probes were developed for the detection of neuro-toxic organophosphonates, such as dimethyl methyl phosphonate (DMMP), by the modification of diminished surfaces with hexafluoro-hydroxypropanyl benzene (HFHPB) as non-covalent sites of interactions (Figure 4). In this manner, different photo-luminescent properties were recorded in the absence and presence of the targeted DMMP on the modified GQDs (HFHPB-GQDs) from their different binding energies collected. 

The chemical modification of GQDs is highly sensitive to their molecular conjugation and media. For example, the best quantum yield was enhanced with four times higher values in modified graphene quantum dots via esterification with benzyl alcohol in optimized media [53]. Moreover, the spectroscopic properties from single GQDs and collective emissions could be modified depending on their individual photo-luminescent properties [54]. The emission spectra on individual GQDs and white light N-doped GQDs (IGQDs) can be prepared by electron beam irradiation with different excitations, such as 488.0 nm, 532.0 nm, and 633.0 nm. In this manner, both forms of single GQDs obtained recorded narrower emissions than from the ensemble depending on their band-to-band transitions and new defect states generated from condensed matter. In a similar manner, variable electronic properties should be mentioned, such as quenched to increased conductions that could be achieved by different states of aggregations and condensed graphene matter interactions [55].

From these tunable single GQDs, varied optical metamaterials could be developed, such as single smart responsive GQDs to metasurfaces, meta-lens, and meta-mirrors by accurate material modifications. In addition, these properties could be joined to other high-energy electromagnetic fields generated at nanoscales. These important optical active approaches could be the platforms from where new matter properties and applications could be generated, for example, the synthesis of chiral GQDs [56] with potential applications of chiral molecular detection on single nanoplatforms towards Van der Walls heterostructures where photons strongly interact. Thus, the assemblies could lead to new metamaterial properties, such as opto-electronic applications [57] and the tuning of plasmonic properties between the interaction of metallic nanoparticles and GQDs. The quantum implications exploiting the Fermi level shift with variations in optical transitions by voltage biasing in the presence and absence of silver nanoparticles should be noted [58]. In this case, the size of the unit cell of study was in the order of 300–500 nm spots depending on the depositions of 0.5 GQDs depositions, and the additions of 45 nm silver nanoparticles with silica spacer intervals of 10–15 nm. This was just an example of tuning plasmonic properties joining different sources of electronic properties and electromagnetic fields. These types of studies could greatly affect the final metamaterial properties depending on the designed approach and optical setups used. So, a broad window of opportunities is shown in this field with a large number of variables to control and the potential generation of new modes of energy and properties. However, there are limitations on the control of chemical surface modifications and associated properties. Even if they showed high sensitivity against passivating agents, modifiers, and colloidal media, in comparison to other semiconductors, improved performances from hybrid quantum, nano-, and micro-materials were achieved. Thus, further developments can join different optical active materials where graphene and derivatives could act as connectors. 

Therefore, challenges to tune from single to dimeric and trimeric and further assemblies within smaller scale lengths are added, and some other important materials could be taken into account. Other carbon-based semiconductors are of interest, such as carbon nanotubes [59], derivatives, and carbon allotropes [60]. In addition, other varied atomic inorganic constitutions of quantum dots based on non-metallic atoms, such as Zn, Cd, Te, Se, etc. [61,62], are of interest in contemplating inorganic matter. In addition, metallic and non-metallic nanoparticles [63], plasmonic materials [64] with variable constitutions, and nanoarchitectures [65], with the incorporation of varied groups of elements from the periodic table, showed impact [66] within photonics and related applications. Explanations of new properties afforded to classical and non-classical models can be applied to new metamaterials [67]. From these varied sources of semiconductive properties, we highlight studies and applications of perovskites for energy and solar cells [68,69]. The efficiency of perovskite solar cells has increased dramatically over the past few years, rising from around 3% in 2009 to over 25% in 2022 [70,71]. In this way, the perovskite chemical structure is known as ABO_3_ (MgSiO_3_, FeSiO_3_, and Al_2_O_3_) from natural sources such as silicate perovskite (MgSiO_3_) [72] to synthetic perovskites with variable atomic constitution showed different chemical structures and crystalline packaging [73]. This variability of chemical conformations and geometries showed a capacity to include variable additional chemical species, atomic defects, ions, etc., that affected structures and properties. In particular, controlled and accurate perovskite chemical structures showed lower energy voltages of band gaps, which opened the study and allowed it to be used for energy applications. For example, the methylammonium halides, the most common of which is methyl ammonium triiodide (CH_3_NH_3_PbI_3_), showed interesting properties for dye-sensitized solar cells related to the generation of free electrons and holes, with consequent improved electronic conductions and diminution of energy losses [74]. In this context, heterojunctions contemplating graphene and related structures are very interesting approaches to design and study.

Thus, optical wires, enhancers, optical couplers, and enhanced hybrid plasmonic properties considering pseudo-electromagnetics from carbon-based materials and intense near-field phenomena from other inorganic materials could be proposed. In a similar manner, other nanoarchitectures based on accurate particle assembling open new optical resonant structures [75]. These new ideas intend to augment the possibilities of developing nano-optical platforms in current research and future projects that contemplate linear and non-linear optics approaches [76]. Varied models should be contemplated to fit experimental data, focusing on the design and the characterization of new materials.

## 4. Discussion about Their Applications in Microdevices and Circuits

Based on the knowledge of tuning variable matter compositions within confined surfaces or volumes, the optical properties could be controlled. In order to accomplish that, the appropriate energy stimulation and source applied using voltage-gated circuits, electronic, magnetic, photonics systems, etc., should be controlled. The optical properties could be reflected and transduced across the surfaces and through the material with modified and improved signaling after the meta-matter interactions. Thus, this post-transduction signaling, depending on the intrinsic constitutive materials, was the targeted study for applications. Thus, varied designs, such as optical nano-platforms, nanodevices, lab-on-a-chip, and microdevices, should be mentioned. 

**Figure 5:**
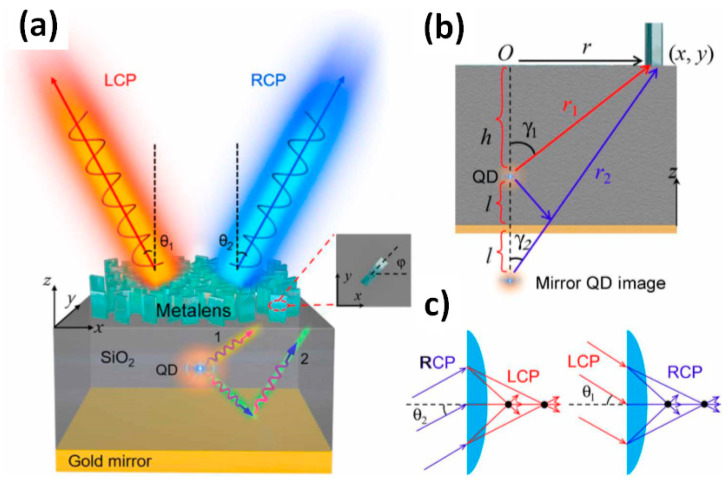
Design of metasurface for on-demand spin-state control of single-photon emission. (**a**) Illustration of the designed structure for manipulating QD emission. The structure consists of three layers: a top metasurface layer, a middle dielectric layer with a QD embedded, and a bottom gold reflector layer. The metasurface is designed to convert the QD emissions from the two paths (labeled 1 and 2) into two opposite circularly polarized beams that propagate along the directions with angles of θ1 and θ2 relative to the surface normal to the metasurface, respectively. The inset shows the top view of a silicon nanoblock with a rotation angle of φ. (**b**) Side view of relative positions of QD and silicon nanoblock. r1 (r2) is the distance from the silicon nanoblock r(x, y) to the QD (mirror QD image), and γ1(γ2) is the angle between the direction r1 (r2) and z-axis. h and l are the vertical distances from the QD to the metasurface and the gold mirror, respectively. (**c**) Schematic of the equivalent two-focus metalense of the metasurface. The LCP and RCP illuminated at incident angles of θ1 and θ2 can be focused into the same two foci. Reprinted with permissions of X. H. Wang et al., 2020 [47], Sci. Adv.

In this manner, focusing on the generation of new properties by the incorporation of graphene within metamaterials, it should be mentioned that different developments highlight varied applications such as photodetectors based on highly sensitive fractal responses and highly sensitive sensors based on improved conductions and piezoelectrics [77]. Regarding sensitivity, microdevices should be taken into account toward the macroscale by their incorporation within textiles and wearable devices [78]. Moreover, the versatility of graphene and derivatives generated different sizes and shapes, such as graphene fibers, nanowires, and grafted structures with high-impact applications within material sciences. In this manner, graphene fibers with predetermined deformation as moisture-triggered actuators and robots [79], current-driven terahertz light emissions [80], and tunable electronic properties in graphene devices [81] should be mentioned. In this way, an accurate material modification could be designed for optical chips based on dielectric metasurfaces by the incorporation of single cold atoms to generate multi-beams of desired polarization states with laser excitations [82]. Moreover, the high sensitivity and accurate state-of-the-art manipulation of the different optical active components within metasurfaces should be highlighted. For example, on-demand spin-state manipulations were controlled on single-photon emissions from quantum dots integrated with metasurfaces (Figure 5) [83]. The mentioned advanced development was permitted by the use of quantum dot manipulations as optical mirrors to generate separation of the spin states of the emitted single photons. These emissions were flexibly manipulated to propagate along a targeted direction with high collimation with divergence angles of 3.17°, including the on-demand manipulation of the polarization, direction, propagation, and collimation of the emitted photon streams. From there, this approach could be extended to other tuneable nanoplatforms, as discussed in the previous section. In this way of thinking and contemplating a broad view of potential uses, the stability of the material obtained by the incorporation of graphene could be considered as hard to receive high-energy stimulations for life science and biosensor applications, as well as their related devices. Further perspectives should be contemplated, such as heterojunctions, where many interesting insights are already published [84]. 

Moreover, there are challenges related to the manipulation of graphene due to aggregation and the lack of solubility if it is required to manipulate colloidal dispersions to develop wet chemical methods with perspectives to be transferred in physics and optics laboratories. From this experimental point of view, it is very important to know that the properties and performances are dependent on the layers contemplating three dimensions. So, as a general rule, single layers do not show the same properties as multi-layered and aggregated highly conjugated carbon-based materials or other inorganic materials. This fact is due to the interaction and modification of close electromagnetic activity; their close surroundings create a different electronic media [85]. Thus, recent developments and emerging applications of graphene-based metamaterials vary by tuning electromagnetics [86]. In this manner, graphene is under focus and offered as a new technology option and, in many cases, incorporated within current technology from the design of nano- and microdevices to the fabrication of higher-sized integrated optoelectronic circuits [87,88,89].

There is potential work for quantum devices exploiting metasurface optical chips for key applications such as sensing, quantum computing, and more with the use of advanced setups [90]. Thus, it was possible to transfer the optical properties generated in controlled setups for research to portable and flexible devices, microlens, textiles, and wearables that enlarged and overlapped the frontiers between the different topics and developments mentioned.

Finally, it should be mentioned that more recent current applications based on optical graphene properties exist for potential uses within the life sciences. For example, the single-stranded deoxyribonucleic acid (ssDNA) detections using graphene resonances from nano-slots with optical active response in the terahertz frequencies after targeted interaction and laser stimulations that permitted biodetection at the nano-mole level [91] and in vivo imaging and drug delivery applications by graphene oxide structures [92]. 

A large number of developments have occurred because of the incorporation of graphene, and many others are in progress. However, other new approaches of basic studies for further properties with potential applications should be proposed in different research fields, even contemplating new ones not developed in the present article, such as quantum biology, bioenergy, bioelectronics, and bio-optics [93].

## Data Availability

Further data related to the development of the present article could be provided upon need by A.G.B.
